# Comparison of bone texture between normal individuals and patients with Kashin-Beck disease from plain radiographs in knee

**DOI:** 10.1038/s41598-018-35552-8

**Published:** 2018-11-30

**Authors:** Wenrong Li, Jukka Hirvasniemi, Xiong Guo, Simo Saarakkala, Mikko J. Lammi, Chengjuan Qu

**Affiliations:** 1 Department of Medical Imaging, The First Affiliated Hospital of Xi´an Jiaotong University, 277 West Yanta Road, Xi´an Shaanxi, 710061 P. R. China; 2 School of Public Health, Xi´an Jiaotong University Health Science Center, Xi´an, P. R. China; 3 Center for Machine Vision and Signal Analysis, Faculty of Information Technology and Electrical Engineering, University of Oulu, Oulu, Finland; 4 Research Unit of Medical Imaging, Physics and Technology, Faculty of Medicine, University of Oulu, Oulu, Finland; 5 Medical Research Center Oulu, Oulu University Hospital and University of Oulu, Oulu, Finland; 6 Department of Diagnostic Radiology, Oulu University Hospital, Oulu, Finland; 7 Department of Integrative Molecular Biology, Umeå University, 90187 Umeå, Sweden

## Abstract

To compare tibial bone texture between Kashin-Beck disease (KBD) patients and normal individuals from plain radiographs using an advanced image analysis. Plain knee radiographs were obtained from KBD patients (n = 49) and age-matched healthy controls (n = 98). KBD were graded with diagnostic criteria WS/T 207-2010. The textural values related to bone structure from medial and lateral tibial subchondral and trabecular bones were evaluated using entropy of Laplacian-based image (E_Lap_), entropy of local binary patterns (E_LBP_), homogeneity indices (HI) of local angles (HI_Mean_, HI_Perp_ and HI_Paral_), and fractal dimensions from horizontal (FD_Hor_) and vertical (FD_Ver_) structures. KBD patients were shorter in height and lighter in weight, and their tibial width was wider than controls. Anatomical angle of KBD patients showed more genu valgus. Total KBD patients and subgroups had higher E_Lap_, HI_Mean_, HI_Perp_ and HI_Paral_ in detected tibial subchondral and trabecular bones than controls, except E_Lap_ in lateral subchondral bone. E_LBP_, FD_Hor_ and FD_Ver_ from the detected tibial bone in KBD patients and subgroups were lower than controls, except FD_Ver_ in lateral trabecular bone. Our results indicate that micro-scale in bone texture in KBD-affected knees can be quantitatively examined from plain radiographs using an advanced image analysis.

## Introduction

Kashin-Beck disease (KBD) is a chronic endemic and degenerative form of osteoarthritis (OA), which is distributed primarily in agricultural regions of southeastern Siberia, northern Korea, and China^1–3^. It causes pain, discomfort and disability to work already at young age. The incidence of KBD has remarkably declined over the last decades, but the disease is still readily observable in the endemic areas, such as Linyou and Yongshou counties at Shaanxi province in China. Despite increasing research on KBD, its etiology remains still unclear.

**Figure 1:**
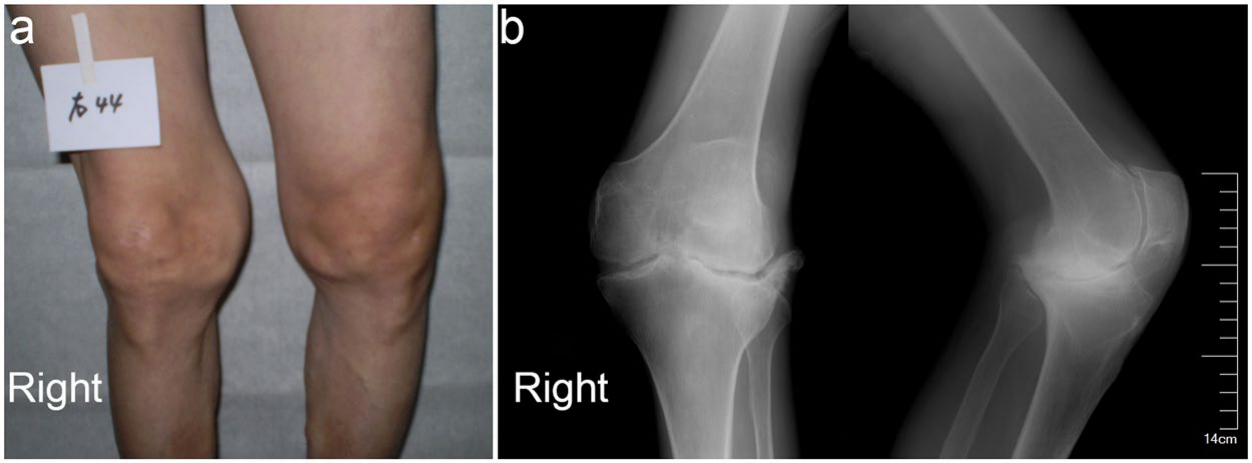
Clinical (**a**) and radiological (**b**) findings in a 55-year old female with KBD in right knee joint.

The KBD’s clinical symptoms are pain, enlargement, morning stiffness, dysfunction, short fingers/limbs and even deformation in the affected joints, which lead to limitations in movements (Fig. 1). Conventional low-cost radiography using a simple and fast two-dimensional (2D) projection has proved to be the best way to diagnose KBD along with patients’ history and symptoms evaluated according to diagnostic criteria WS/T 207–2010 (Supplemental Table 1)^1,4^. The characteristic features of KBD in plain radiography are blurred, interrupted and irregular marginal sclerosis in epiphysis and/or metaphysis^5,6^. Macro-scale changes of bone tissues can be easily observed from plain radiographs which provide very useful morphological information of the disease-affected bone density and structure. However, these changes are difficult to visually evaluate from plain radiographs due to flat image and overlapping anatomical structures, as compared to three-dimensional (3D) imaging.

Radiologists often diagnose bone disease using trabecular bone microarchitecture. However, the anatomy and mechanical properties of trabecular bone are not easy to observe precisely due to the shape and distribution of trabecular networks. Trabecular bone microstructure from 2D radiographs and local binary patterns (LBP) -based methods^7^ for plain radiographs have been used for OA studies. It has been reported that bone density or/and structure, evaluated from 2D radiographs of different bones using these techniques, are strongly correlated with true 3D microstructure in normal and diseased human individuals^8–13^. Bone texture in normal and osteoarthritic knees has been quantitatively evaluated from 2D radiographs using Laplacian-based and LBP-based images^7,10,14^, which showed that bone structure-related parameters had significant differences between different OA grades, and between healthy control and OA groups^7,15^.

To our knowledge, there are no any studies on bone texture analysis from 2D radiography in KBD patients yet. Therefore, we aimed to quantify whether the differences in bone structure between healthy individuals and KBD patients, and within various grades of KBD patients can be revealed from 2D radiographs using bone texture analysis methods. We hypothesized that bone structure-related texture parameters measured with Laplacian-based, LBP-based and FSA-based methods are different between healthy individuals and KBD patients or their subgroups.

## Results

Height, weight and body mass index (BMI) were smaller in total KBD patients than the age-matched controls, as did the height in KBD grades 2 and 3, as well weight in all KBD sub-groups (Table 1, Supplemental Table 1). Width of the tibia in total KBD patients and KBD grades 2 and 3 was larger than the controls, while anatomical angle in KBD grade 1 and 3 was larger than the controls, showing genu valgus (Table 2). MedJSW in KBD grade 3 was lower than the controls and KBD grade 1, as well as it was also lower in KBD grade 2 than grade 1 (Table 2).

**Table 1:** Basic information of the subjects used in the present study.

Parameter		Mean ± SD					Statistical significance
Subjects (KBD grades)		Control (0)	KBD total (KBD)	KBD grade 1 (1)	KBD grade 2 (2)	KBD grade 3 (3)	
Age (years)		51.2 ± 8.6	52.1 ± 10.7	51.7 ± 12.2	54.1 ± 8.2	48.4 ± 11.3	none
Height (cm)		161.0 ± 6.8	155.5 ± 9.8	158.0 ± 9.0	154.2 ± 7.6	150.4 ± 15.1	0-KBD**, 0–2*, 0–3*
Weight (kg)		59.0 ± 8.3	52.7 ± 9.4	54.7 ± 10.3	51.8 ± 8.5	48.0 ± 7.0	0-KBD**, 0–1*, 0–2**, 0–3**
Body mass index (kg/m^2^)		22.7 ± 2.6	21.7 ± 2.7	21.8 ± 2.9	21.7 ± 2.3	21.3 ± 2.8	0-KBD*
Gender	Female Male	70 28	32 17	17 7	11 7	4 3	

Normal control subjects, n = 98; total KBD patients, n = 49; KBD grade 1, n = 24, KBD grade 2, n = 18 and KBD grade 3, n = 7. SD: standard deviation; KBD: Kashin-Beck disease; **stands for p value is equal or less than 0.001, *represents that p value is less than 0.05.

**Table 2:** Mean values (±SD) of medJSW, latJSW, tibia width and anatomical angle of the knees from the subjects.

Parameter	Mean ± SD					Statistical significance
Subjects (KBD grades)	Control (0)	KBD total (KBD)	KBD grade 1 (1)	KBD grade 2 (2)	KBD grade 3 (3)	
medJSW	4.22 ± 0.9	3.96 ± 1.2	4.38 ± 0.8	3.71 ± 1.3	3.15 ± 1.3	0–3*, 1–2*, 1–3*
latJSW	4.03 ± 1.0	4.15 ± 1.8	4.24 ± 1.8	4.19 ± 2.1	3.76 ± 0.8	none
Tibia width	73.18 ± 5.1	77.09 ± 7.6	75.17 ± 7.0	79.80 ± 8.2	76.69 ± 6.7	0-KBD**, 0–2**, 0–3*
Anatomical angel, genu valgus	182.11 ± 2.9	183.60 ± 7.0	183.78 ± 4.1	182.92 ± 10.5	184.71 ± 3.1	0–1*, 0–3*
	−2.11 ± 2.9	−3.60 ± 7.0	−3.78 ± 4.1	−2.92 ± 10.5	−4.71 ± 3.1	0–1*, 0–3*

Normal control subjects, n = 98; total KBD patients, n = 49; KBD grade 1, n = 24, KBD grade 2, n = 18 and KBD grade 3, n = 7. SD: standard deviation; KBD: Kashin-Beck disease; medJSW: minimum joint space width measured from 75% medial compartment; latJSW: minimum joint space width measured from 75% lateral compartment; Genu varus/valgus were calculated with the equation: 180° - anatomical angle; **stands for p value is equal or less than 0.001, *represents that p value is less than 0.05.

**Figure 2:**
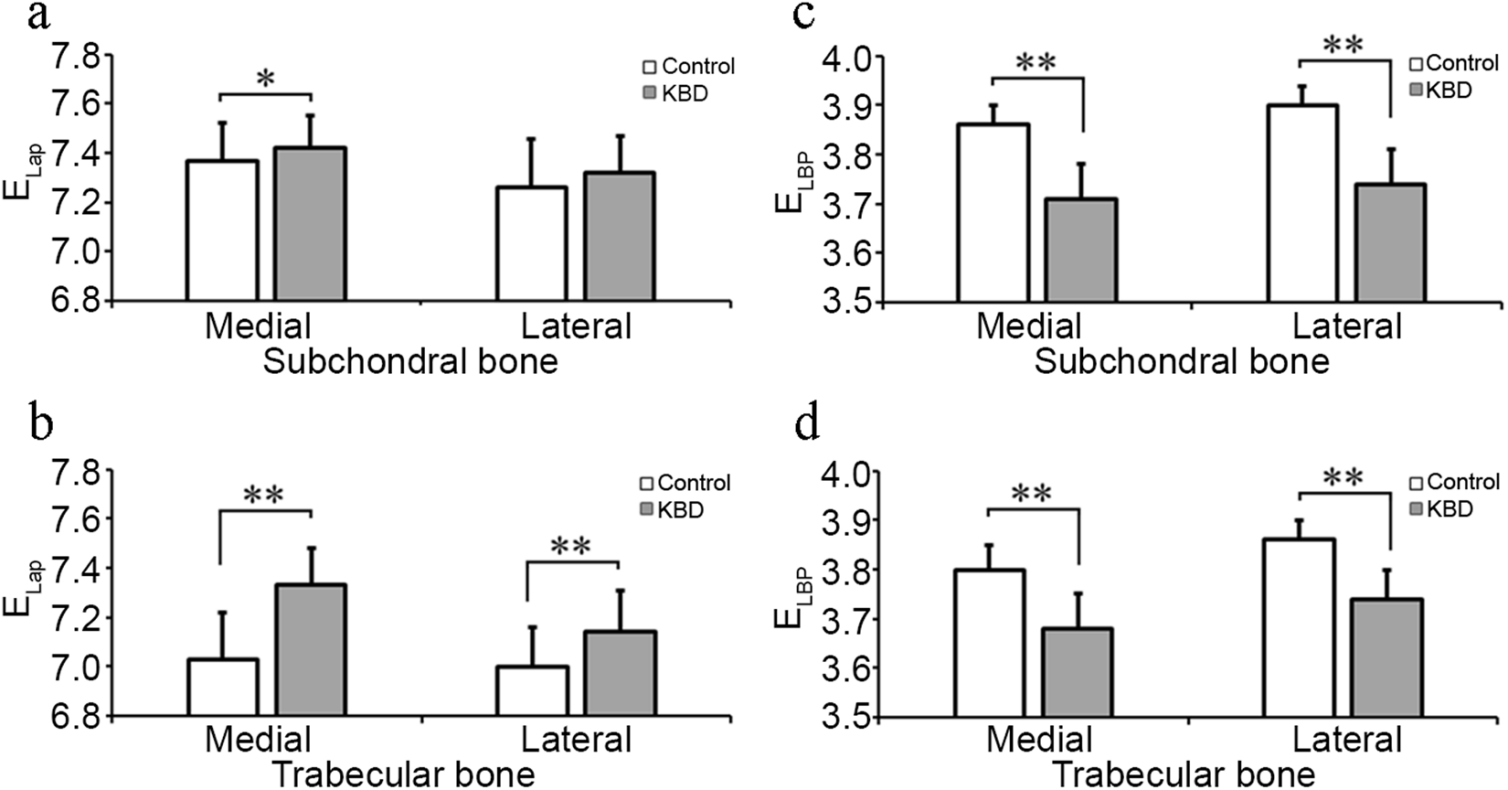
Representative mean ± SD and statistical significance of bone structural parameters, entropy of laplacian-based image (E_lap_) and local binary patterns (E_LBP_) measured from medial and lateral subchondral and trabecular bone between control healthy individuals (*n* = 98) and Kashin-Beck disease (KBD, *n* = 49) patients. *Stands for P < 0.05, **Stands for P ≤ 0.001.

**Figure 3:**
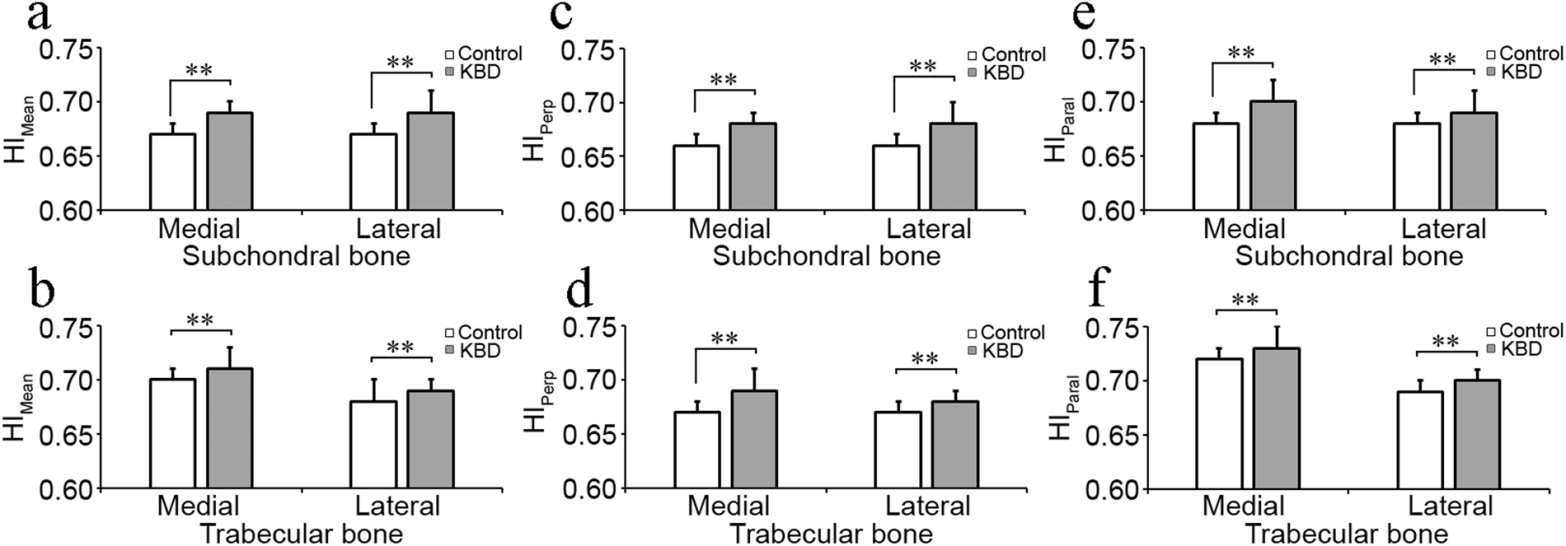
Representative mean ± SD and statistical significance of bone structural parameters, mean of homogeneity index (HI_Mean_) and HI perpendicular (0°, HI_Perp_) and parallel (90°, HI_Paral_) to the trabecular main orientation, measured from medial and lateral subchondral and trabecular bone between control individuals (*n* = 98) and Kashin-Beck disease (KBD, *n* = 49). *Stands for P < 0.05, **Stands for P ≤ 0.001.

**Figure 4:**
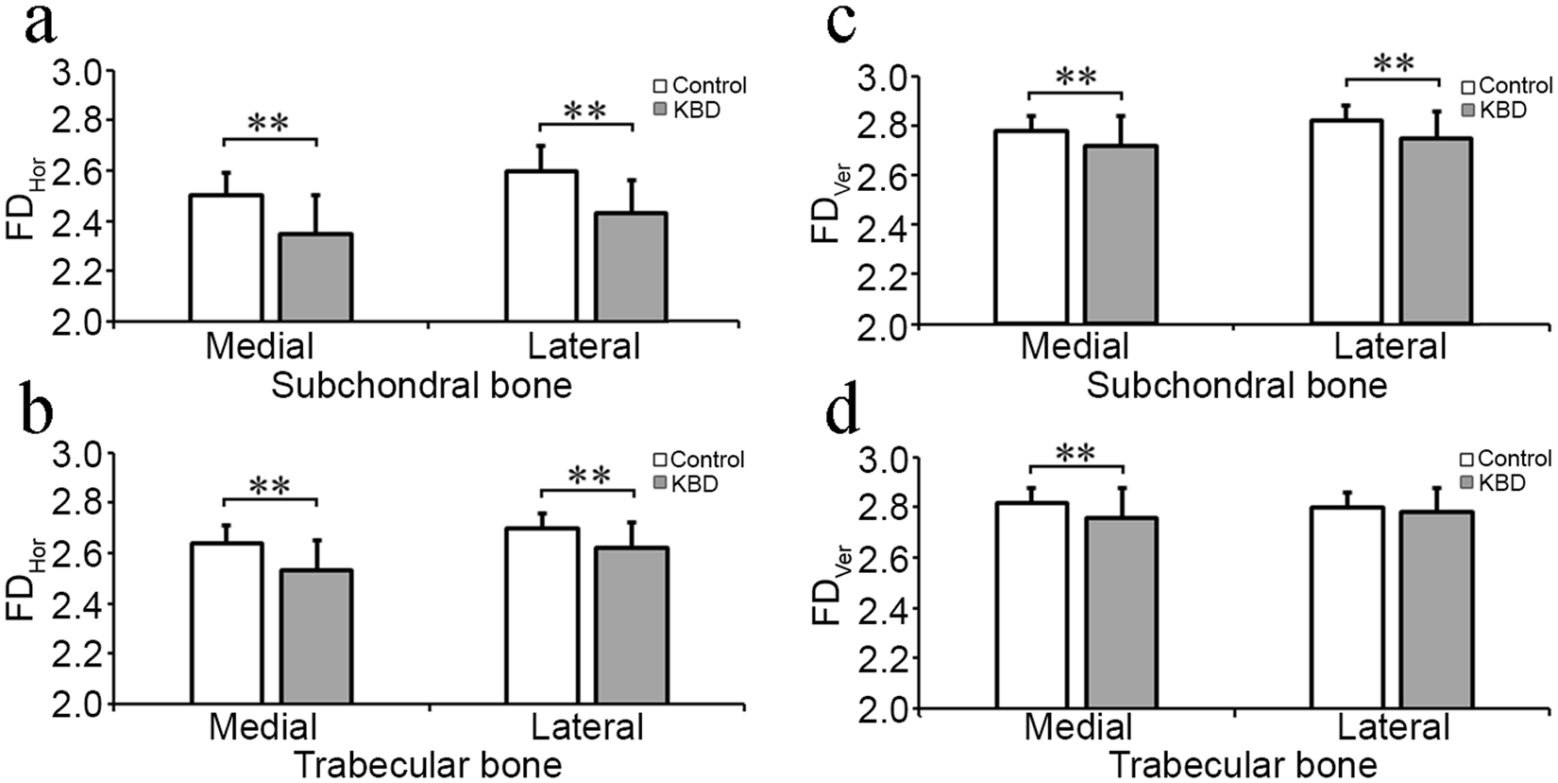
Representative mean ± SD and statistical significance of bone structural parameters, fractal dimension of horizontal (FD_Hor_) and vertical structures (FD_Ver_), measured from medial and lateral subchondral and trabecular bone between control individuals (*n* = 98) and Kashin-Beck disease (KBD, *n* = 49). *Stands for P < 0.05, **stands for P ≤ 0.001.

Statistically significant differences were observed in bone texture parameters. In medial subchondral bone, E_Lap_ in total KBD patients and all KBD subgroups was higher than the controls, as well as it was lower in KBD grades 2 and 3 compared to grade 1 (Fig. 2a, Table 3). HI_Mean_, HI_Perp_ and HI_Paral_ were higher in total KBD patients, as well in various KBD subgroups than the controls (Fig. 3a,c,e, Table 3). Furthermore, HI_Mean_ and HI_Paral_ were also higher in KBD grades 2 and 3 than grade 1 (Table 3). However, E_LBP_, FD_Hor_ and FD_Ver_ were lower in total KBD patients and various KBD subgroups than the controls (Figs 2c, and 4a,c, Table 3).

**Table 3:** Mean values (±SD) of the measurement related to the bone structural parameters in control (n = 98) and KBD (n = 49) subjects from medial and lateral subchondral bone plate in tibia.

Side of joint	Parameter	Control (0)	KBD total (KBD)	KBD grade 1 (1)	KBD grade 2 (2)	KBD grade 3 (3)	Statistical significance
Medial subchondral bone	E_Lap_	7.37 ± 0.15	7.42 ± 0.13	7.46 ± 0.12	7.37 ± 0.14	7.44 ± 0.06	0–KBD*, 0–1*, 0–2*, 0–3*, 1–2*, 1–3*
	E_LBP_	3.86 ± 0.04	3.71 ± 0.07	3.72 ± 0.08	3.70 ± 0.06	3.70 ± 0.06	0–KBD**, 0–1**, 0–2**, 0–3**
	HI_Mean_	0.67 ± 0.01	0.69 ± 0.01	0.69 ± 0.01	0.70 ± 0.01	0.71 ± 0.02	0–KBD**, 0–1**, 0–2**, 0–3**, 1–2*, 1–3*
	HI_Perp_	0.66 ± 0.01	0.68 ± 0.01	0.68 ± 0.01	0.69 ± 0.01	0.69 ± 0.02	0-KBD**, 0–1**, 0–2**, 0–3**
	HI_Paral_	0.68 ± 0.01	0.70 ± 0.02	0.69 ± 0.01	0.71 ± 0.01	0.71 ± 0.02	0–KBD**, 0–1**, 0–2**, 0–3**, 1–2*, 1–3*
	FD_Hor_	2.50 ± 0.09	2.35 ± 0.15	2.39 ± 0.16	2.33 ± 0.14	2.30 ± 0.12	0-KBD**, 0–1**, 0–2**, 0–3**
	FD_Ver_	2.78 ± 0.06	2.72 ± 0.12	2.74 ± 0.09	2.69 ± 0.12	2.71 ± 0.17	0–KBD**, 0–1*, 0–2**, 0–3*
Lateral subchondral bone	E_Lap_	7.26 ± 0.20	7.32 ± 0.15	7.37 ± 0.10	7.26 ± 0.16	7.33 ± 0.20	0–1*, 0–2*, 0–3*, 1–2*
	E_LBP_	3.90 ± 0.04	3.74 ± 0.07	3.77 ± 0.07	3.72 ± 0.08	3.74 ± 0.07	0–KBD**, 0–1**, 0–2**, 0–3**, 1–2*
	HI_Mean_	0.67 ± 0.01	0.69 ± 0.02	0.68 ± 0.01	0.69 ± 0.02	0.69 ± 0.02	0–KBD**, 0–1**, 0–2**, 0–3**
	HI_Perp_	0.66 ± 0.01	0.68 ± 0.02	0.67 ± 0.02	0.68 ± 0.01	0.68 ± 0.01	0–KBD**, 0–1**, 0–2**, 0–3**
	HI_Paral_	0.68 ± 0.01	0.69 ± 0.02	0.69 ± 0.01	0.70 ± 0.02	0.70 ± 0.02	0–KBD**, 0–1**, 0–2** 0–3**
	FD_Hor_	2.60 ± 0.10	2.43 ± 0.13	2.46 ± 0.13	2.43 ± 0.14	2.39 ± 0.06	0–KBD**, 0–1**, 0–2**, 0–3**
	FD_Ver_	2.82 ± 0.06	2.75 ± 0.11	2.77 ± 0.10	2.73 ± 0.12	2.72 ± 0.11	0–KBD**, 0–1*, 0–2**, 0–3**

SD: standard deviation; KBD: Kashin-Beck disease; E_lap_: entropy of Laplacian-based image; E_LBP_: entropy of local binary patterns; HI_mean_: homogeneity index for orientation of local patterns; HI_perp_; HI perpendicularly to the bone trabeculae; HI_paral_: HI parallel to the bone trabeculae; FD_hor_: fractal dimension of horizontal structures; FD_ver_: fractal dimension of vertical structures. no: no statistical significance. **Stands for p value is equal or less than 0.001, *represents that p value is less than 0.05.

In lateral subchondral bone, E_Lap_ was higher in various KBD subgroups than the control, as well as it was higher in KBD grade 1 than grade 2, but no significant difference was observed on E_Lap_ between controls and total KBD patients (Fig. 2a, Table 3). HI_Mean_, HI_Perp_ and HI_Paral_ were higher in total KBD patients as well in various KBD subgroups than the controls (Fig. 3a,c,e, Table 3). E_LBP_ , FD_Hor_ and FD_Ver_ were lower in total KBD patients as well as in various KBD subgroups than the controls (Figs 2c and 4a,c, Table 3). Moreover, E_LBP_ in KBD grade 2 was lower than grade 1 (Table 3).

In medial trabecular bone, E_Lap_, HI_Mean_, HI_Perp_ and HI_Paral_ were higher in total KBD patients and various KBD subgroups than the controls (Figs 2b and 3b,d,f, Table 4), as well as HI_Mean_ and HI_Perp_ were higher in KBD grades 2 or 3 than grade 1 (Table 4). E_LBP_ , FD_Hor_ and FD_Ver_ in total KBD patients and various KBD subgroups were lower than the controls (Figs 2d, and 4b,d, Table 4). Furthermore, E_LBP_ was lower in KBD grade 2 than grade 1 (Table 4), and FD_Ver_ was lower in KBD grades 2 and 3 than the controls (Table 4).

**Table 4:** Mean values (±SD) of the measurement related to the bone structural parameters in control (n = 98) and KBD (n = 49) subjects from medial and lateral trabecular bone in tibia.

Side of joint	Parameter	Control (0)	KBD total (KBD)	KBD grade 1 (1)	KBD grade 2 (2)	KBD grade 3 (3)	Statistical significance
Medial trabecular bone	E_Lap_	7.03 ± 0.19	7.33 ± 0.15	7.31 ± 0.16	7.35 ± 0.15	7.34 ± 0.18	0–KBD**, 0–1**, 0–2**, 0–3**
	E_LBP_	3.80 ± 0.05	3.68 ± 0.07	3.70 ± 0.06	3.66 ± 0.07	3.68 ± 0.11	0–KBD**, 0–1**, 0–2**, 0–3**, 1–2*
	HI_Mean_	0.70 ± 0.01	0.71 ± 0.02	0.71 ± 0.01	0.72 ± 0.01	0.71 ± 0.03	0–KBD**, 0–1**, 0–2**, 0–3**, 1–2*
	HI_Perp_	0.67 ± 0.01	0.69 ± 0.02	0.69 ± 0.01	0.70 ± 0.01	0.69 ± 0.03	0–KBD**, 0–1**, 0–2**, 0–3**, 1–2*, 1–3*
	HI_Paral_	0.72 ± 0.01	0.73 ± 0.02	0.72 ± 0.02	0.73 ± 0.01	0.73 ± 0.02	0–KBD**, 0–1*, 0–2**, 0–3**
	FD_Hor_	2.64 ± 0.07	2.53 ± 0.12	2.56 ± 0.12	2.49 ± 0.11	2.51 ± 0.11	0 – KBD**, 0–1**, 0–2**, 0–3**
	FD_Ver_	2.82 ± 0.06	2.76 ± 0.12	2.79 ± 0.08	2.71 ± 0.11	2.76 ± 0.21	0 – KBD**, 0–2**, 0–3**, 1–2*, 1–3*
Lateral trabecular bone	E_Lap_	7.00 ± 0.16	7.14 ± 0.17	7.12 ± 0.18	7.15 ± 0.17	7.21 ± 0.15	0–KBD**, 0–1*, 0–2**, 0–3**
	E_LBP_	3.86 ± 0.04	3.74 ± 0.06	3.76 ± 0.04	3.71 ± 0.05	3.72 ± 0.08	0–KBD**, 0–1**, 0–2**, 0–3**, 1–2**, 1–3*
	HI_Mean_	0.68 ± 0.01	0.69 ± 0.01	0.69 ± 0.01	0.70 ± 0.01	0.69 ± 0.02	0–KBD**, 0–1*, 0–2**, 0–3**, 1–2*, 1–3*
	HI_Perp_	0.67 ± 0.01	0.68 ± 0.01	0.68 ± 0.01	0.69 ± 0.01	0.68 ± 0.01	0–KBD**, 0–1**, 0–2**, 0–3**, 1–2*
	HI_Paral_	0.69 ± 0.01	0.70 ± 0.01	0.70 ± 0.01	0.71 ± 0.01	0.71 ± 0.02	0–KBD**, 0–2**, 0–3**, 1–2*, 1–3*
	FD_Hor_	2.70 ± 0.06	2.62 ± 0.10	2.64 ± 0.10	2.60 ± 0.10	2.58 ± 0.10	0–KBD**, 0–1**, 0–2**, 0–3**
	FD_Ver_	2.80 ± 0.06	2.78 ± 0.10	2.80 ± 0.09	2.75 ± 0.09	2.78 ± 0.13	none

SD: standard deviation; KBD: Kashin-beck disease; E_lap_: entropy of Laplacian-based image; E_LBP_: entropy of local binary patterns; HI_mean_: homogeneity index for orientation of local patterns; HI_perp_; HI perpendicularly to the bone trabeculae; HI_paral_: HI parallel to the bone trabeculae; FD_hor_: fractal dimension of horizontal structures; FD_ver_: fractal dimension of vertical structures. **Stands for p value is equal or less than 0.001, *represents that p value is less than 0.05.

In lateral trabecular bone, E_Lap_, HI_Mean_, HI_Perp_ and HI_Paral_ were higher in total KBD patients and various KBD subgroups than the age-matched controls, besides HI_Mean_ and HI_Paral_ were higher in KBD grades 2 and 3 than grade 1, and HI_Perp_ was higher in KBD grade 2 than grade 1 (Figs 2b, and 3b,d,f, Table 4). However, E_LBP_ and FD_Hor_ in total KBD patients and various KBD subgroups were lower than the controls, as well as E_LBP_ was lower too in KBD grade 2 and 3 than grade 1 (Figs 2d, and 4b, Table 4). However, no significant difference could be seen on FD_Ver_ between KBD groups and the controls (Table 4).

## Discussion

Our results revealed that the diseased subjects had lower height, weight, anatomical femur-tibia angle and medJSW, but wider tibia, confirming that the patients investigated here suffered from KBD^1^. The most interesting findings in this study are that the bone structure measured from medial and lateral tibial subchondral and trabecular bones from 2D radiographs showed statistical differences in KBD patients and/or their subgroups when compared to controls, which shows in following parameters: E_Lap_, E_LBP_ , HIs, and FDs. Interestingly, the changes of the bone texture parameters in KBD are mostly opposite to OA patients when compared to controls, suggesting that micro-structural changes in bone are not identical to OA (Supplemental Table 2).

E_Lap_ is used to examine randomness of the pixel in an image, whereas E_LBP_ measures randomness of local binary patterns^7,15,16^. When E_Lap_ is equal to zero, the image is perfectly flat and pixel intensities are identical within image. Lower E_Lap_ indicates lower variation in the image pixel intensities. However, when E_LBP_ is equal to zero, it means that there is only one pattern occurring in the analyzed image. Lower E_LBP_ value indicates lower number of the grouped LBPs in the analyzed image. In this study, inverse relationship between E_Lap_ and E_LBP_ was observed in KBD similarly to OA, although in an opposite direction. Therefore, the increased E_Lap_ and decreased E_LBP_ indicates that KBD have more variation in grayscale values in the detected bones than controls, but less variation in different LBPs in the image. Since our studied subjects are mainly mild and moderate KBD cases, the less disorganized different patterns in KBD may act as remodeling of the bone structure and keep the balance for local disoriented pattern before developing to OA, even though the increased E_LBP_ noticed in advanced OA^7^. In KBD, the paramount histopathologic changes are chondrogenesis, secondary repair and tissue remodeling, and they may happen separately or even simultaneously^6^.

The higher HI, the more connected trabecular fibers are in an image. The more similar orientation in the adjacent patterns, the closer to one the value of HI will be. Thus, the variation in various directions of local patterns would be low in that case. Our results with higher HI_Mean_, HI_Perp_ and HI_Paral_ in KBD, but less than one, indicated that the orientation of neighbouring patterns is more similar to the local ones, and more connected to the main trabecular fibers, as the trabeculae are more vertically aligned than horizontally. This is further confirmed with the increased E_lap_ and the decreased E_LBP_ in this study, which showed better connectivity of bone structure and the adjacent patterns differed less from each other in KBD, but opposite to advanced OA (Supplemental Table 2)^7,15^.

FDs were calculated locally in vertical and horizontal directions with FSA method, which provides the most precise characteristics of roughness of trabecular bone texture^17,18^. FSA has been suggested as a potentially efficient mean to predict OA progression and help on the treatment and research^19^. A significantly lower FD_Hor_ and FD_ver_ in KBD in this study indicated the thickening of the trabeculae in horizontal and vertical directions in both sides of the tibia, and may cause trabecular sclerosis similarly to the findings observed in KBD hand radiographs^1,6^. A loss of bone and bone structure from both sides appears obvious, although no significant changes observed in FD_ver_ in lateral trabecular bone. These are consistent with previous studies on OA knees with Kellgren & Lawrence (KL) grade 2 or worse, which showed significantly lower FDs in OA knees than controls (Supplemental Table 2)^20,21^. However, this finding is inconsistent with previous results of lower E_Lap_ and HIs, and higher E_LBP_ and FDs on KL grade 2 or worse OA^15^. One explanation for the contradicting results is that the KBD recruited for this study were mostly at mild and moderate disease stage. Moreover, the reactive bone proliferation and disturbance of bone formation are the secondary changes in KBD besides the primary changes on cartilage^6^. Another explanation is that KBD patients had lower BMI whereas OA typically have higher BMI than controls.

This study contains certain limitations that should be addressed. First, the imaging settings were not completely consistent for the subjects, which might have affected image quality and bone texture parameters. To avoid this factor, same equipment, imaging parameters and calibration object in the image should be used in future studies. To overcome this issue in this study, the images were resampled to a same pixel size, median filtered, and grayscale value range was expanded to full dynamic range. Texture parameter are robust to change in magnitude of grayscale values in image. Second, due to different diagnostic criteria used for KBD and OA, the comparison of their bone texture parameters showed a difference. Finally, the imaging findings, such as cysts and sclerosis, can also affect bone texture. Therefore, it would be advantageous to use same radiographic criteria to diagnose KBD and OA in order to make effective comparison.

## Conclusion

The bone texture structure in KBD had clearly changed in subchondral and trabecular bone. The Laplacian-based, LBP-based, and FSA methods are powerful tools to quantify micro-scale changes of KBD in bone texture anisotropy, randomness and roughness. These analyses can be easily performed with a low-cost 2D digitalized high-resolution radiographs. The findings on micro-changes of anisotropy, connectivity and roughness underlying subchondral bone could play a key role in the initiation and progression of KBD knee joints. The incidence of KBD is high during age 5–15 years old, and hands are most frequently affected. Hand radiographs are usually the first choice to diagnose and classify KBD. The developed FSA methods have been successfully used to evaluate trabecular bone texture in OA knee and hand radiographs^22,23^. Therefore, our future work on hand bone texture analysis could provide wider knowledge for early stage diagnosis of KBD and young patients, and prevention and treatment.

Plain radiographs has been utilized as a general examination method in the diagnostic of KBD, which can provide very useful information on the morphological changes of the affected bone density and structure at macro-scale. Thus, it has been used as a first diagnostic tool in radiology for KBD. However, the microarchitecture changes in subchondral and trabecular bone in KBD or other bone disease are not easy to quantify reliably in the basic plain radiographs with visual evaluation. Therefore, the bone texture structure analysis with Laplacian-based, LBP-based and FSA methods would provide more quantitative data of the micro-scale changes of KBD patients in bone texture anisotropy, randomness and roughness, and may help in the clinical diagnostic of the disease at early stage.

## Methods

### Study materials

A total of 116 controls and 77 KBD patients were primarily recruited in the study. Of these, 18 controls and 28 KBD patients were excluded in the study due to the quality of the plain radiographs and/or the presence of osteophytes, which would affect the measurements. Thus, 98 controls and 49 KBD patients were included in the analysis of the present study. 98 healthy individuals (age: 51.2 ± 8.6; 28 male and 70 female) with 98 plain radiographs were randomly selected from non-endemic area, which was close to the endemic area, and had similar environment concerning geography, climate and life style as the endemic KBD area. 49 KBD patients (age: 52.1 ± 10.7; 17 male; 32 female) with 49 plain radiographs were selected from endemic disease areas. Written informed consent was obtained from all studied subjects and/or their legal guardians with permission to be used as their personal background information in the study. This study was reviewed and approved by “The first Affiliated Hospital of Xi´an Jiaotong University Ethics Committee”, and the project implementation process was in line with the ethical principles.

### Acquisition of plain radiographs and grading of knees

Right knees of control (*n* = 98) and 46 right and three left knees of KBD patients (*n* = 49) were imaged at posterior-anterior fixed–flexion position using digital radiographies with tube voltage: 55–65 kV, quantity of charge: 200 mA, exposure time: 12–20 ms and source-detector distance: 85 cm. The KBD patients were graded based on the diagnostic criteria of clinical appearance (http://www.moh.gov.cn/zwgkzt/s9500/201006/47920.shtml, Supplemental Table 1). For grading, value one means mild KBD, while three indicates severe KBD (Table 1). All the imaged knees were analyzed with a custom-made MATLAB software (v7.9.0; MathWorks Inc., Natick, MA, USA). In order to get reliable measured parameters, all images were rescaled to a pixel size of 150 µm adjusted to achieve a full dynamic range and converted to 8-bit before analyses.

### Selection of regions of interest

**Figure 5:**
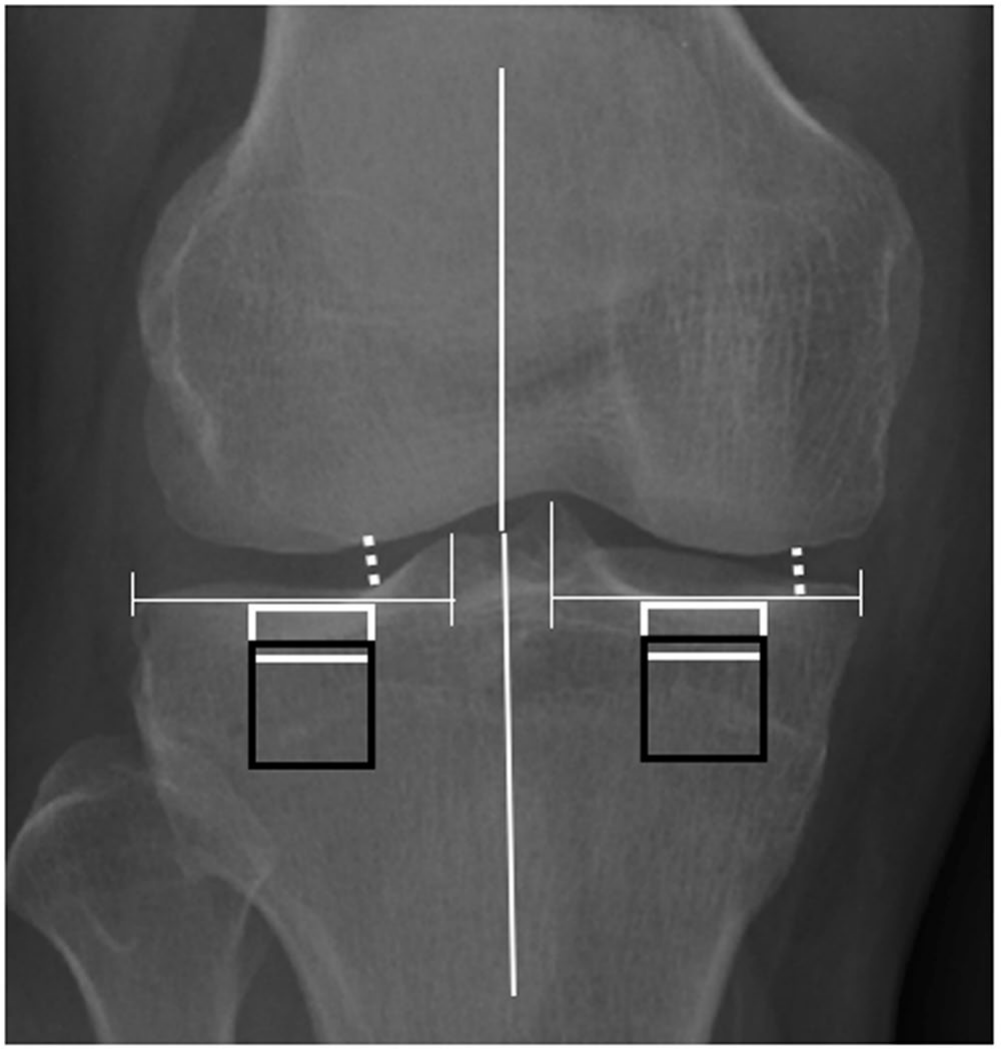
Schematic figure of placement ROIs. Two subchondral bone ROIs (93 × 40 pixels, white rectangles) were placed under the cartilage-bone interface in the middle part of medial and lateral condyles of the tibia. Two trabecular bone ROIs (93 × 93 pixels, black rectangles) were placed below and parallel to the subchondral bone ROIs of the tibia. Minimum JSWs (white dotted line) were measured from the narrowest point of the joint from both the medial and the lateral sides. Anatomical angles were medially measured from the intersection of a line from the center of the head of femur to the center of the tibial spines, and a second line from the center of the tibia to the center of the tibial spines.

As previous studies described^7,15^, two ROIs (93 × 40 pixels) subchondral bone were placed in the center of medial and lateral condyle of tibia below cartilage-bone interface, and two other ROIs (93 × 93 pixels) for trabecular bone were closely placed below and parallel to the dense subchondral bone. The minimum medial (medJSW) and lateral joint space widths (latJSW) were also measured in the narrowest point of joint from both medial and lateral sides as previously described^7^. The width of tibia was measured from the corners of both sides of the bone. The anatomical angle was measured based on a gold standard criterion in a full limb radiograph, where mechanical-axis angle (180°) was used as reference standard, with angles <180° defined as genu varus, and angles >180° defined as genu valgus^24^ (Fig. 5).

### Analysis of bone texture

The structure of bone texture was analyzed using Laplacian-based, LBP-based^7,10,14–16^, and FSA-based methods^10,18^ after the radiographs were median-filtered (3 × 3 pixels) to eliminate the high frequency noise from the images.

#### Laplacian-based analysis

Laplacian-based method has been clearly shown to promote the appearance of bone trabeculae and quantitatively determine the variations in the grayscale values on Laplacian-based images^14^. As previous studies described in Laplacian-based analysis^7,14,15^, the Laplacians were calculated in both vertically and horizontally, then combined into one matrix. A final Laplacian-based image was obtained after an unprocessed ROI was multiplied with square root of Laplacian matrix and further expanded to a full dynamic range. The entropy of image (E_Lap_) was used to present the randomness of grayscale values in Laplacian-based image^7,14,15^ calculated with following equation 1:

1 $${{\rm{E}}}_{{\rm{Lap}}}=-\,{\sum }_{i}{{\rm{P}}}_{i}\,\mathrm{log}\,2\,{{\rm{P}}}_{i},$$

where E_Lap_ is the entropy of a statistical textural characteristic of randomness, and P_*i*_ is the normalized grayscale value *i* in the image.

#### Local binary patterns (LBP)-based methods

LBP, a 2D texture spectrum model on pixel level in grayscale, have been a powerful feature for texture classification in computer vision field^25,26^. It encodes the relative strength of the center pixel and its surrounding pixels, which represents eight elements. Each of those has one of two possible values (0, 1) obtained from a neighborhood of 3 × 3 pixels. The center pixel is set up as threshold and compares to 8-neighborhood pixel. LBP-based methods were used to evaluate the randomness of local binary patterns of the images and the dissimilarities in the orientation of adjacent local binary patterns as described elsewhere in previous studies^10,15,27^. Briefly, the bone and non-bone areas of the image was determined using Otsu method and LBPs were calculated from these regions. The number of possible patterns was reduced by determining the main orientation of each pattern. The orientation angles (0°, 45°, 90°, and 135°) were computed for the patterns consisting of two to five consecutive markers. Rest of the patterns were considered as non-uniform. The entropy of the grouped patterns (E_LBP_) determined with equation (1) was used to present the randomness of the patterns in an image.

#### Homogeneity index (HI) for orientation of local patterns

HI, a spatial distribution of gray-level co-occurrence matrix in an image, evaluates the variations in neighboring pattern orientations along the defined direction. The co-occurrence matrices of local pattern angles were computed at 0°, 45°, 90°, and 135° directions within one pixel’s distance. HI-derived from the gray-level co-occurrence matrix was calculated perpendicular (HI_Perp_, combination of 45° and 90°) and parallel (HI_Paral_, combination of 0° and 135°) to the trabecular main orientation^15^. The mean of HI (HI_Mean_) was the sum of the four possible directions calculated from the co-occurrence matrices.

#### Fractal signature analysis (FSA)

FSA is used to quantify degree and content of the roughness of bone texture structure in an image as previously described^10,15,28,29^. In practice, the original image is firstly dilated and eroded in vertically and horizontally with a structuring element that is rod-shaped and one-pixel-wide^15,18^, and whose length varied from 2 to 4 pixels. The volume measured between the dilated and eroded image and the structuring element length were used to calculate surface area, which was made in log-log plot and then together with structuring element length, were used to estimate FD by using a regression line to points. When the structuring element is pointing in vertical direction, FD of horizontal structures (FD_Hor_) is produced, similarly, the horizontal direction produces FD of vertical structure (FD_Ver_)^15,18,30^.

### Statistical analysis

Statistical analyses were performed using IBM SPSS statistics 24 software. The differences of structure-related parameters between controls and KBD patients, and various KBD subgroups were evaluated with non-parametric independent Mann-Whitney U/Kruskal Wallis H sample test. *P* value of <0.05 was considered as statistical significance.

### Statistics and biometry

Jukka Hirvasniemi kindly provided statistical advice for this manuscript.

### Informed consent

Written informed consent was obtained from all subjects (patients) in this study.

### Ethical approval

Institutional Review Board approval was obtained from “The first Affiliated Hospital of Xi´an Jiaotong University Ethics Committee”.

### Study subjects or cohorts overlap

Study subjects or cohorts have not been previously reported.

### Methodology

Retrospective, cross sectional study, performed at one institution. All methods were carried out in accordance with relevant guidelines and regulations.

## Electronic supplementary material

Supplemental Table 1 and Table 2

## Data Availability

The original, raw data and quantitative data considering the basic information, medJSW, latJSW, tibia width and anatomical angle of the knees, bone structural parameters (E_Lap_, E_LBP_ , HI, FD) from subchondral and trabecular bone of the studied subjects used to support the findings of this study are available from corresponding author upon reasonable request. The images for patient’s clinical and radiological findings as well as the placement of ROIs used to support the findings of this study are available from corresponding author upon request.
