# Supplementary material for: Comparison of bone texture between normal individuals and patients with Kashin-Beck disease from plain radiographs in knee
Source: Sci Rep. 2018 Nov 30;8:17510. doi: 10.1038/s41598-018-35552-8 (PMC6269488; doi:10.1038/s41598-018-35552-8)
Supplement: Supplementary file 1 — Supplemental Table 1 and Table 2 [file 41598_2018_35552_MOESM1_ESM.pdf]

Comparison of bone texture between normal individuals and patients with Kashin-Beck disease from plain radiographs in knee

Wenrong Li[1,2] Jukka Hirvasniemi[3] Xiong Guo[2]* Simo Saarakkala[4] Mikko J. Lammi[2,5] Chengjuan Qu[5]*

[1] Department of medical imaging, the First Affiliated Hospital of Xi´an Jiaotong University, 277 West Yanta Road, Xi´an Shaanxi, 710061, PR China (WR.L.)

[2] School of Public Health, Xi´an Jiaotong University Health Science Center, Xi´an, PR China

[3] Center for Machine Vision and Signal Analysis, Faculty of Information Technology and Electrical Engineering, University of Oulu, Oulu, Finland

[4] Research Unit of Medical Imaging, Physics and Technology, Faculty of Medicine, University of Oulu, Oulu, Finland; Medical Research Center Oulu, Oulu University Hospital and University of Oulu, Oulu, Finland; Department of Diagnostic Radiology, Oulu University Hospital, Oulu, Finland

[5] Department of Integrative Molecular Biology, Umeå University, 90187 Umeå, Sweden

Address correspondence to *Chengjuan Qu:
chengjuan.qu@gmail.com; chengjuan.qu@umu.se

Supplemental Table 1 The clinical and X-ray diagnostic criteria of Kashin-Beck disease (KBD) (WS/T 207-2010).

Criterion	KBD	Description
Clinical diagnosis	Stage I	Multiple, symmetrical enlarged finer joints, limited flexion and extension, pain and mild muscle atrophy.
	Stage II	Based on stage I, clinical symptoms aggravated, and appeared shortened fingers.
	Stage III	Based on stage II, clinical symptoms aggravated and appeared short limbs and dwarfism.
X-ray diagnosis	Stage I (Mild)	If have one of the flowing conditions<br>a). Only metaphyseal lesion, +<br>b). Lesion only in the end of bone, +<br>c). Lesions in both of talus and calcaneus, +
	Stage II (Moderate)	If have one of the following conditions<br>a). Only metaphyseal lesion, ++<br>b). Lesion only in the end of bone, ++<br>c). Lesions in both metaphysis and end of bone<br>d). Lesions in both epiphysis and metaphysis<br>e). Lesions in both carpal and end of bone<br>f). Talus lesion, ++
	Stage III (Severe)	If have one of the following conditions<br>a). Metaphyseal lesion, +++<br>b). Lesion in the end if the bone, +++<br>c). Lesions in metaphysis, end of bone, epiphysis and/or carpal (three out of four locations or in all four)<br>d). Metaphyseal premature closure<br>e). Lesions in both of talus and calcaneus, +++

Supplemental Table 2 Comparison of tibial bone structure-related parameters between osteoarthritis (OA) from previous four studies and Kashin-Beck disease (KBD) in our present study.

Bone structure-related parameters	Subchondral bone in OA		Subchondral bone in KBD		Trabecular bone in OA		Trabecular bone in KBD	
	Medial	Lateral	Medial	Lateral	Medial	Lateral	Medial	Lateral
E_{Lap}	-** (ref 7);<br>-* (ref 15)	no(ref 7)	+*	no	no (ref 7);<br>-* (ref 15)	-* (ref 7)	+**	+**
E_{LBP}	+**(ref 7);<br>+ *(ref 15)	no(ref 7)	-**	-**	+**(ref 7);<br>+* (ref 15)	+** (ref 7)	-**	-**
HI_{Mean}	-* (ref 15)		+**	+**	-* (ref 15)		+**	+**
HI_{Perp}	-**(ref 15)		+**	+**	-* (ref 15)		+**	+**
HI_{Paral}	no (ref 15)		+**	+**	-* (ref 15)		+**	+**
FD_{Ver}	+**(ref 15)		-**	-**	+* (ref 15)<br>-**(ref 20)<br>-**(ref 21)	-* (ref 20);<br>-** (ref 21)	-**	no
FD_{Hor}	no (ref 15)		-**	-**	+*(ref 15);<br>-* (ref 20);<br>-**(ref 21)	-* (ref 20);<br>-** (ref 21)	-**	-**

Osteoarthritis: OA; KBD: Kashin-Beck disease; E_{lap}: entropy of Laplacian-based image; E_{LBP}: entropy of local binary patterns; HI_{mean}: homogeneity index for orientation of local patterns; HI_{perp}; HI perpendicularly to the bone trabeculae; HI_{paral}: HI parallel to the bone trabeculae; FD_{hor}: fractal dimension of horizontal structures; FD_{ver}: fractal dimension of vertical structures. "-"means decreased compared to control; "+" means increased compared to control; no: no statistical significance; ** stands for p value is less than 0.001, * represents that p value is less than 0.05. ref 7: reference Hirvasniemi et al. 2014; ref 15: reference Hirvasniemi et al. 2017; ref 20: Podsiadlo et al. 2008; ref 21: Wolski et al. 2010.
